# Physicians’ knowledge, attitudes, and perceptions concerning antibiotic resistance: a survey in a Ghanaian tertiary care hospital

**DOI:** 10.1186/s12913-018-2899-y

**Published:** 2018-02-20

**Authors:** Appiah-Korang Labi, Noah Obeng-Nkrumah, Stephanie Bjerrum, Nii Armah Adu Aryee, Yaw Adjei Ofori-Adjei, Alfred E. Yawson, Mercy J. Newman

**Affiliations:** 1 Department of Microbiology, Korle-Bu Teaching Hospital, P.O. Box 77, Accra, Ghana; 2 Department of Medical Laboratory Sciences, University of Ghana School of Biomedical and Allied Health Sciences, P.O Box 143, Korle-Bu Accra, Ghana; 3 Department of Infectious Diseases, Copenhagen University Hospital, Rigshospitalet, Blegdamsvej 9, 2100 Copenhagen, Denmark; 4 Department of Surgery, University of Ghana School of Medicine and Dentistry, P.O. Box 4326, Accra, Ghana; 5 Department of Medicine, Korle-Bu Teaching Hospital, P.O. Box 77, Accra, Ghana; 6 Department of Community Health, School of Public Health, College of Health Sciences, University of Ghana, Accra, Ghana; 7 Department of Medical Microbiology, School of Biomedical and Allied Sciences, P.O. Box KB, 143 Accra, Ghana

**Keywords:** Views, Antibiotic resistance, Antibiotics, Physicians, Ghana, Korle-Bu Teaching Hospital

## Abstract

**Background:**

Understanding the knowledge, attitudes and practices of physicians towards antibiotic resistance is key to developing interventions aimed at behavior change. The survey aimed to investigate physicians’ knowledge and attitudes towards antibiotic resistance in a tertiary-care hospital setting in Ghana.

**Methods:**

We conducted a cross-sectional respondent-driven survey using a 40-item, anonymous, voluntary, traditional paper-and-pencil self-administered questionnaire among 159 physicians at Korle-Bu Teaching Hospital. Single and multi-factor analysis were conducted to assess the study objectives.

**Results:**

The survey was completed by 159 of 200 physicians (response rate of 79.5%). Of physicians, 30.1% (47/156) perceived antibiotic resistance as very important global problem, 18.5% (29/157) perceived it as very important national problem and only 8.9% (14/157) thought it as a very important problem in their hospital. Methicillin resistant *Staphylococcus aureus* was the most known about antibiotic resistant bacteria of public health importance followed by extended-spectrum beta-lactamase-producing *Enterobacteriaceae*, carbapenem resistant *Enterobacteriaceae* (CRE) and vancomycin resistant enterococci (VRE). In multiple logistic regression analysis, senior physicians were nearly 3 times more likely to know about CRE than junior physicians. The odds of knowing about VRE increased over 4.5 times from being a junior to becoming senior physician. Among junior physicians, age had no associated effect on their knowledge of VRE or CRE.

**Conclusions:**

Physicians in this survey showed variable knowledge and perceptions on antibiotic resistance. Introducing educational programs on antibiotic resistance would be a useful intervention and should focus on junior physicians.

**Electronic supplementary material:**

The online version of this article (10.1186/s12913-018-2899-y) contains supplementary material, which is available to authorized users.

## Background

Antibiotics are among the most commonly used drugs in hospitals and the community [1]. The effectiveness of antibiotics in preventing and treating infections is threatened by the global rise in bacteria resistance [2, 3]. Addressing the challenge of antibiotic resistance involve efforts aimed at modifying behaviour of physicians towards prescription of antibiotics [4–6]. This requires understanding of the physicians’ knowledge, attitudes, and practices (KAP) towards antibiotic resistance. It has been suggested that physicians are likely to change their antibiotic prescribing behavior when their knowledge, attitudes and skills are aligned to the reduction of antibiotic resistance [7]. Several KAP surveys on antimicrobial resistance have been conducted among physicians in community settings [8–13]. Fewer studies have been performed among physicians in tertiary care settings, and these are mostly from Europe [11, 14, 15] and the Americas [10, 16]. In Africa, KAP studies on antibiotic resistance are rare [17–20]. In all these studies, the authors have consistently identified gaps in the knowledge, attitudes, and perceptions of physicians towards antibiotic resistance which are important to promote the rational use of antibiotics and develop interventions aimed at behavior change [15].

Most surveys that have assessed KAP of physicians on antibiotic resistance involved  univariate analyses. To the best of our knowledge, only one study has conducted multivariate analysis to investigate factors that affect physicians’ KAP on antibiotic resistance [21]. These findings however may not necessarily be applicable in our setting as physicians’ perceptions of antimicrobial resistance have been shown to vary across institutions [14]. In Ghana, studies have shown high levels of antibiotic resistance in hospitals [22–25], but published data assessing the KAP of physicians towards antibiotic resistance are absent. In this study, we have used univariate comparisons and multiple regression analysis to assess the knowledge, attitudes and perceptions of physicians on antibiotic resistance in a tertiary care setting. To report our findings, we followed guidelines for Strengthening the Reporting of Observational Studies in Epidemiology for respondent-driven sampling studies: “STROBE-RDS” statement [26].

## Methods

### Study design and setting

We conducted a cross-sectional respondent-driven study among physicians of the Korle-Bu Teaching Hospital (KBTH) in August 2015. The Korle-Bu Teaching Hospital is a 2000 bed referral facility serving the Ghanaian population as well as some countries in the West African sub-region. In 2013, the central outpatient department at Korle-Bu Teaching Hospital recorded about 30,000 patient consultations per month; with over 138 admissions per day [27].

### Study population

Physicians of all levels of expertise at the Departments of Medicine, Surgery, Paediatrics and Obstetrics and Gynaecology were purposefully selected to complete self-administered questionnaires. These departments have the highest number of patient consultations per day and the largest number of physicians attached to the hospital. In a count of permanent physicians on the nominal roll of the selected hospital departments, 200 physicians in total were found, and all were considered eligible to participate in our study. They included house officers (qualified doctors practicing under supervision in the first year after graduation), medical officers/residents (medical graduates engaged in specialized practice under supervision), senior residents/specialists, and senior specialist/consultants.

### Survey instrument

The questionnaire (Additional file 1) used in this study was developed in consultation with experts on infectious diseases, and after searching the literature for similar studies [15, 17, 28]. The questionnaire was English-based; and subjected to pre-testing with 5 physicians to check for clarity and comprehension of questions. The survey instrument covered questions on knowledge, attitude and perception. Data on the practices of physicians’ regarding antibiotic resistance will be published elsewhere. The 40-item self-administered questionnaire collected information under four cluster headings. The first included demographics and general information data (including age, gender, level of training, years of practice experience after completion of medical school, and the department of practice). The second cluster comprised perceptions on the importance of the antibiotic resistance problem (including perceived importance of the problem at the global, national, hospital, and at the unit/departmental level of practice; impact of the antibiotic resistance on patients’ safety at the department of practice; and the self-reported experience of the inappropriate use of antibiotics in the department of practice). The third collected information was about perceptions on causes of antibiotic resistance. On the basis of review of literature [15, 17, 28], we selected eight possible causes of antibiotic resistance including self-medication, uncompleted antibiotic therapy, use of antibiotics in humans, and poor quality antibiotics. Here, participants were asked to rate the extent to which those factors contribute to the development of antibiotic resistance. The last cluster incorporated questions on knowledge and attitude on antibiotic resistant bacteria of public health importance [including knowledge and management of infections by methicillin resistant *Staphylococcus aureus* (MRSA), extended-spectrum beta-lactamase-producing *Enterobacteriaceae* (ESBL), carbapenem resistant *Enterobacteriaceae* (CRE), and vancomycin resistant enterococci (VRE)]. Questions on perceptions of antibiotic resistance used 3-point Likert-style response options from very important to minimally important. To assess knowledge on the prevalence of antibiotic resistant bacteria of public health importance, physicians were asked to choose the correct order of prevalence for MRSA, ESBL, CRE, and VRE. The KBTH has a higher prevalence of infections caused by ESBL [24, 29] compared to MRSA [30, 31]. It is also anecdotal experience that CRE are encountered more often than VRE, but there are no published reports to document this evidence. Thus any order of prevalence for the resistant bacteria ESBL>MRSA>VRE > CRE or ESBL>MRSA> CRE > VRE was considered to correct.

### Survey administration

The survey questionnaires were used to collect data over a 2-week period. Data collection was led by the principal investigator at post ward-round clinical meetings to maximize the chances of reaching physicians in the selected departments. Completed questionnaires were collected at the end of the day. Questionnaires not returned within 1 week triggered personal reminders. There was no incentive for physicians to participate. Each questionnaire included a cover letter explaining the purpose of the study and a consent form requesting the participation of physicians and assuring that confidentiality would be maintained. The questionnaires were without identifiers to ensure anonymity of respondents.

### Statistical methods

Statistics on baseline characteristics of the study hospital including local guidelines, and the prevalence of CRE and VRE were obtained with assistance from the hospital’s Research and Development Unit. Study data was analyzed with the Statistical Package for Social Sciences Version 21 (IBM, USA). Proportions were calculated for categorical variables; means and standard deviations were calculated for continuous variables. We used the chi-square test for comparison of categorical data, or Fisher’s exact test when needed. Unadjusted odds ratio (OR) at 95% confidence interval (95%CI) with chi-square test was used to estimate univariate associations. Variables with statistical significance at *P* < 0.05 were incorporated into a multivariate logistic regression models to identify knowledge, attitudes or perceptions that are independently associated with being a senior or junior physician. Junior physicians referred to doctors from house officers to those still in their residency training. Post residency trained doctors were considered senior physicians. Physician variables that were not retained in the models by this procedure were tested for confounding by adding them one at a time and examining their effects on the ß coefficients. Variables that caused substantial confounding (change in ß coefficient of 110%) were included in the final model. Predictive accuracy of the models was assessed by Hosmer and Lemeshow goodness-of-fit test with *P* > 0.05 indicating that the model predicts accurately on average. The area under the Receiver Operating Characteristic (ROC) curve was used to evaluate the discriminatory capability of the models. Effect size determination was then used to compare significant terms from the multivariate logistic analysis — with possible interaction of the variables computed with analysis of covariance (ANCOVA) [21]. Collinearity was examined with the tolerance statistics by replacing variables with each other and examining the effect on the models.

## Results

### Demographics

The survey was completed by 159 of 200 physicians (response rate of 79.5%) from 4 specialties. The mean age of respondents was 32.11 ± 6.52 years (Table 1). Respondents included 64 (40.31%) house officers, 52 (32.73%) medical officers/residents, 33(20.82%) senior residents/specialists, and 10 (6.30%) senior specialist/consultants. After dichotomizing the 4-option level of training, 72.90% (116/159) and 27.0% (43/159) of the sample comprised junior and senior medical physicians respectively. A total of 74 (46.51%) respondents had > 5 years practice experience, whiles 19 (11.90%) had practiced for 3–5 years, 6 (3.80%) for 1–2 years, and 60 (37.71%) for < 1 year.

**Table 1:** Baseline characteristics of hospital and physicians participating in the study

Hospital characteristics	Description	Number of respondents	Percentage
Type of hospital	2000 bed university public teaching hospital	159	–
Prevalence of infections caused by ESBL-producers [24]	Data retrieved from a cross-sectional study	350	44.3
Prevalence of MRSA infections [31]	Data retrieved from a cross-sectional study		3.0
Prevalence of CRE infections	Data not published	250	0.3
Prevalence of VRE infections	Data not published	100	0
Availability of local antibiotic guidelines	No		
Availability of drug and therapeutic committee	Yes	–	–
Access to microbiologist advice	Yes. Mainly through request for clinical consultation, but also some advice by phone; only during working hours		
Access to clinical pharmacists advice in KBTH	Yes. Mainly through request for clinical consultation, but also some advice by phone; only during working hours		
Access to computer-based prescription	No		
Hospital departments	Surgery	26	16.35
	Genitourinary	4	2.51
	Medical	45	28.30
	Obstetrician and Gynecology	25	15.71
	Orthopedics	5	3.14
	Paediatrics	37	23.27
	Other department^b^	17	10.69
Number of physicians surveyed	Of all levels of expertise and training	159	79.50
Age of study participants (mean ± SD)^a^	–	159	32.11 ± 6.52 years
Gender of physicians surveyed	Male	93	58.49
	Female	63	39.6
Level of training among study participants	House officers	64	40.25
	Medical officers/residents	52	32.70
	Senior residents/specialists	33	20.75
	Senior specialists /consultants	10	6.28
Years of practice among study participants	< 1 year	60	37.77
	1–2 years	6	3.77
	3–5 years	19	11.90
	> 5 years	74	46.54

*VRE* vancomycin resistant enterococci, *CRE* carbapenem resistant enterococci, *ESBL* extended-spectrum beta-lactamases, *MRSA* methicillin resistant *Staphylococcus aureus*

^a^SD, standard deviation

^b^Others include polyclinic (*n* = 1), neurosurgery (n = 1), not stated (*n* = 15)

### Perceived problem of antibiotic resistance

Of physicians, 30.11% (47/156) perceived antibiotic resistance as a very important global problem, 18.5% (29/157) a very important national problem, 8.91% (14/157) a very important problem in their hospital, and 5.50% (9/154) a very important problem in their departments (Table 2). Two-thirds of respondents (103/156) perceived the impact of antibiotic resistance on patients’ safety in their departments as very important — this perception was influenced by the belief that antibiotics were used inappropriately in the departments (23/103 versus 19/49; *X*^*2*^ = 10.10; OR, 3.21; *P* = 0.001).

**Table 2:** Perceptions and knowledge of physicians on antibiotic resistance

Physicians’ perceptions and knowledge	Number of respondents (%)			
Perceptions	Very important	Important	Not important	Don’t know
Grade the level of antibiotic resistance worldwide	47(30.1)	67(42.9)	6(3.8)	36 (23.1)
Grade the level of antibiotic resistance in Ghana	29 (18.4)	76 (48.4)	5 (3.2)	47 (29.9)
Grade the level of antibiotic resistance in your hospital	14 (8.9)	81 (51.5)	16 (10.1)	46 (29.3)
Grade the level of antibiotic resistance in your department	9 (5.8)	56 (36.1)	42 (27.1)	48 (30.9)
Rate the impact of antibiotic resistance on patient safety in your department	103 (66.0)	51 (32.7)	2 (1.3)	0
		Yes	No	
Do you think antibiotics are used appropriately in your department?		110 (72.3)	42 (27.6)	
Knowledge	ESBL	MRSA	CRE	VRE
Do you know about the following resistant bacteria?	86 (62.7)	150 (98.6)	157 (37.6)	74 (48.6)
Have you ever managed patients with infections by the following resistant bacteria?	27 (17.3)	49 (31.8)	7 (4.4)	6 (3.9)
Are patients in the hospital at risk of the following resistant bacteria?	78 (51.3)	133 (86.3)	48 (30.5)	67 (43.2)
Does the hospital have a problem with the following resistant bacteria?	38 (24.8)	73 (47.1)	18 (11.5)	15 (9.4)
What is the extent of the hospital’s problem with the following resistant bacteria?				
Very serious	6 (15.7)	25 (34.2)	3 (16.6)	4 (26.6)
Serious	25 (65.7)	37 (50.6)	13 (72.2)	10 (66.6)
Not serious	7 (18.4)	11 (15.1)	2 (11.1)	1 (6.6)
Indicate the correct order of prevalence for the following resistant bacteria in Ghana	E > M > V > C^a^	M > E > C > V	E > M > C > V	Don’t know
All respondents	17 (10.8)	52 (33.3)	10 (6.4)	77 (49.4)
Senior physicians	9 (21.4)	8 (19.1)	5 (11.9)	20 (47.6)
Junior physicians	8 (7.0)	44 (38.5)	5 (4.3)	57 (50.0)
> 5 years of experience	11 (14.6)	21 (28.0)	5 (6.6)	38 (50.6)
< 5 years of experience	6 (7.5)	31 (38.8)	5 (6.25)	38 (47.5)

*ªE* Extended-spectrum Beta-lactamases, *M* Methicillin resistant *Staphylococcus aureus*, *C* carbapenem resistant *Enterobacteriacaea*, *V* Vancomycin resistant Enterococci

### Perceptions on causes of antibiotic resistance

**Fig. 1:**
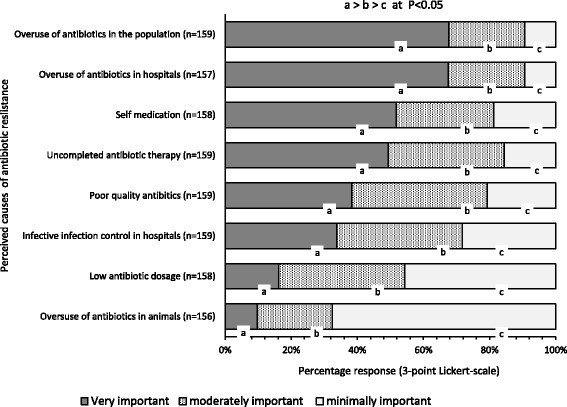
Perceptions of causes of antibiotic resistance. Four factors were identified as being the most important causes of antibiotic resistance: overuse of antibiotics in the population overuse of antibiotics in hospitals, self-medication, and uncompleted antibiotic therapy

Four factors were significantly perceived by the majority of physicians as very important causes of antibiotic resistance (Fig. 1): overuse of antibiotics in the population (69.81%, *n* = 111/159), overuse of antibiotics in hospitals (67.52%, *n* = 106/157), self-medication (55.19%, *n* = 85/154), and uncompleted antibiotic therapy (50.31%, *n* = 80/159). Less than half of the physicians (37.73%, *n* = 60/159) considered poor quality antibiotics and low antibiotic dosage (17.08%, *n* = 27/158) as very important causes of antibiotic resistance. Overuse of antibiotics in animals was perceived as the least important cause of antibiotic resistance (67.94%, n = 106/156). We incorporated the four most perceived very important causes of antibiotic resistance into a multivariable linear model to determine the best predictor of the physician’s’ perception that antibiotic resistance is a very serious problem in Ghana. The latter was best predicted by the perception that antibiotic overuse in hospitals is a very important cause of antibiotic resistance (r = 0.54, *P* = 0.019) — the other three variables in the model were non-significant.

### Knowledge of antibiotic resistant bacteria of public health interest

**Fig. 2:**
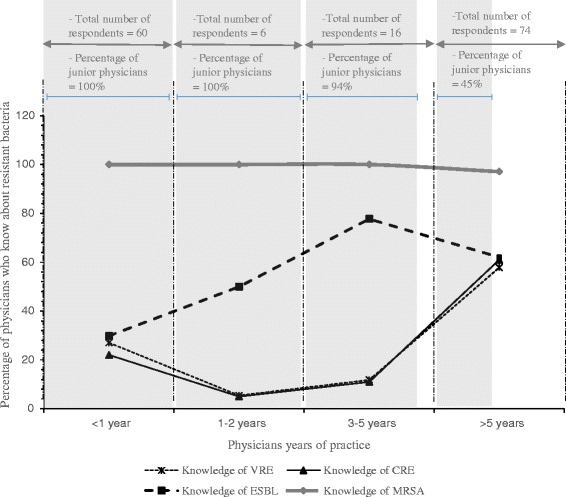
Physicians’ years of practice versus their knowledge of MRSA, ESBL, VRE and CRE. Shaded and white background represent percentage of junior and senior physicians respectively within each category. Chi-square for linear trend (increasing): Knowledge of ESBL, P = 0.001; knowledge of MRSA, P = 0.971; knowledge of knowledge of VRE, P = 0.004; knowledge of CRE, *P* = 0.001. MRSA, methicillin resistant *Staphylococcus aureus*; ESBLs, extended-spectrum beta-lactamases; CRE, carbapenem resistant *Enterobacteriaceae*; VRE, vancomycin resistant *Enterococcus* species

Of the four bacteria groups presented to respondents (Table 2), MRSA (98.74%, *n* = 157/159) and CRE (37.11%, *n* = 59/159) were the most and least known antibiotic resistant bacteria respectively. We therefore evaluated knowledge of ESBL, MRSA, CRE, and VRE versus years of practice and how this varied among junior and senior physicians (Fig. 2). For ESBL, the results can be interpreted as knowledge increasing from 29.80% for physicians who had practiced for < 1 year (junior physicians only) to 62.0% among physicians with > 5 years of practice (55% senior physicians, 45 junior physicians) (overall chi-square test for increasing linear trend, *P* = 0.001). A flat trend was observed for knowledge of MRSA which remained high (> 97%) among physicians with < 1 year of experience through to those with > 5 years of practice (Chi-square tests for increasing linear trend, *P* = 0.971). In contrast, knowledge of CRE and VRE were uncommon among physicians until after 3–5 years of practice (Chi-square for increasing linear trend, *P* = 0.004 for VRE and *P* = 0.001 for CRE).

### Clinical experience versus knowledge of antibiotic resistant bacteria

We sought to examine the responses of physicians’ who knew about ESBL, MRSA, CRE, and VRE and how this related to their clinical experience. Physicians who knew about ESBL were more likely to have previously managed patients with such infections (OR, 1.42; 95%CI, 0.09–3.51; *P* = 0.021); this association was influenced by the physician’s level of training (i.e. senior vs junior doctor: 52% of senior physicians who knew about ESBL had also managed ESBL infections compared to 21% of junior physicians who knew about ESBL and had managed ESBL infections; *X*^*2*^ = 1.91, *P* = 0.001). Similarly, physicians who knew about VRE were more likely to have previously managed patients with such infections (OR, 1.91; 95%CI, 1.11–2.98; P = 0.001) and this association was also influenced by their level of training (i.e. 54% of senior physicians who knew about VRE had previously managed VRE infections compared to 25% of junior physicians who knew about VRE and had managed VRE infections; *X*^*2*^ = 1.34, *P* = 0.019). Physicians who knew about CRE were also more likely to have managed patients with such infections (OR, 1.20; 95%CI, 0.81–1.72; *P* = 0.015) and this association was influenced by their level of training (i.e. 63% of senior physicians who expressed knowledge of CRE had also managed CRE infections compared to 33% of junior physicians who knew about CRE and had also managed CRE infections; *X*^*2*^ = 2.94, *P* = 0.009).

### Respondents characteristics associated with being a senior versus junior physician

Our finding that physicians’ knowledge and perception patterns on antibiotic resistance stratify across level of training (i.e. senior and junior physicians) led us to perform multiple linear regression with the aim of identifying independent physician’s characteristics significantly associated with being a senior or junior physician. In univariate analysis (Table 3), several knowledge patterns regarding antibiotic resistant bacteria of public health importance were noted to be significantly associated with being a senior physician, including knowledge of VRE (OR, 2.71; 95% CI, 1.26–5.81; P = 0.009), CRE (OR, 3.77, 95% CI, 1.79–7.93; *P* = 0.001), and ESBL (OR, 3.57, 95% CI, 1.63–7.85; P = 0.001). Table 4 shows the multivariate analysis of respondents’ variables independently associated with senior physicians. Overall, higher mean age (*P* = 0.01) was independently associated with being a senior doctor. Senior physicians were 2.75 times more likely to know about CRE than junior physicians [adjusted odds ratio (AOR), 2.75; 95% CI, 1.18–4.93; *P* = 0.012]. The results also show that the odds of VRE knowledge increases over 4.5 times from being a junior to becoming senior physician (AOR, 4.63, 95% CI, 1.51–8.22; *P* = 0.001).

**Table 3:** Univariate analysis of physicians’ knowledge of multidrug resistant bacteria of public health importance across senior and junior physicians

Physicians characteristics	Numbers	Level of training		Unadjusted odds ratio	*P*-value
		Senior physicians (*n* = 43)	Junior physicians (*n* = 116)		
Gender (*n* = 156)		42	114		
	Male (*n* = 93)	27	66	1.31 (0.6–2.7)	0.471
	Female (*n* = 63)	15	48		
Age of respondents (*n* = 159)		39.4 ± 6.5	29.3 ± 4.0	2.71 (1.04–5.02)	0.001
Level of antibiotic resistance in hospital (n = 157)		42	115		
	Very important (*n* = 14)	4	10	1.11 (0.33–3.73)	1
	Important (*n* = 81)	26	55	1.77 (0.86–3.65)	0.118
	Not Important (*n* = 16)	3	13	0.60 (0.16–2.23)	0.561
	Don’t know(*n* = 46)	9	37	0.57 (0.25–1.32)	0.191
Level of antibiotic resistance in unit (*n* = 154)		74	80		
	Very important (n = 9)	6	3	2.26 (0.55–9.41)	0.313
	Important (*n* = 56)	30	26	1.41 (0.73–2.74)	0.300
	Not important (*n* = 42)	15	27	0.50 (0.24–1.04)	0.061
	Don’t know(*n* = 48)	23	24	1.05 (0.53–2.09)	0.887
Level of antibiotic resistance in Ghana (n = 157)		42	115		
	Very important (*n* = 29)	5	24	0.51 (0.18–1.44)	0.200
	Important (*n* = 76)	24	52	1.61 (0.79–3.29)	0.185
	Not important (*n* = 7)	2	3	1.86 (0.30–11.59)	0.610
	Don’t know(*n* = 47)	11	36	0.78 (0.35–1.72)	0.381
Level of antibiotic resistance worldwide (n = 156)		41	115		
	Very important(*n* = 103)	30	73	1.26 (0.59–2.69)	0.543
	Important(*n* = 51)	13	38	0.86 (0.40–1.83)	0.689
	Not important (n = 2)	0	2	–	1
Antibiotics used appropriately in department (*n* = 152)		40	112		
	Yes(*n* = 110)	29	81	1.01 (0.45–2.26)	1.00
	No (n = 42)	11	31		
Knowledge of VRE (n = 152)		113	39		
	Yes (*n* = 74)	48	26	2.71 (1.26–5.81)	0.009
	No(*n* = 78)	65	13		
Have ever managed VRE (*n* = 155)		115	40		
	Yes(n = 6)	3	3	3.03 (0.59–15.65)	0.339
	No(*n* = 149)	112	37		
Extent of VRE problem in hospital (*n* = 143)		115	41		
	Very serious(n = 4)	3	1	0.93 (0.09–9.23)	1
	Serious(n = 16)	11	5	1.31 (0.43–4.03	0.764
	Not serious(*n* = 136)	101	35	0.81 (0.29–2.27)	0.689
Knowledge of CRE (n = 157)		116	41		
	Yes (n = 59)	34	25	3.77 (1.79–7.93)	0.001
	No (*n* = 98)	82	16		
Have ever managed CRE (*n* = 158)		116	42		
	Yes (n = 7)	5	2	1.11 (0.21–5.95)	1
	No (*n* = 151)	111	40		
Extent of CRE problem in hospital (*n* = 18)		9	9		
	Very serious (*n* = 3)	2	1	0.44 (0.03–5.93)	1
	Serious (n = 7)	3	4	1.6 (0.24–10.81)	1
	Not serious (n = 8)	4	4	1 (0.16–6.42)	1
Knowledge of ESBLs (n = 152)		111	41		
	Yes (n = 78)	48	30	3.57 (1.63–7.85)	0.001
	No(n = 74)	63	11		
Have ever managed ESBLs (n = 156)		114	42		
	Yes (n = 27)	11	16	5.76 (2.39–13.89)	0.001
	No (*n* = 129)	103	26		
	No(*n* = 115)	89	26		
Extent of ESBL problem in hospital (*n* = 38)		23	15		
	Very serious (n = 6)	4	2	0.73 (0.11–4.59)	1
	Serious (*n* = 25)	15	10	1.06 (0.26–4.22)	0.921
	Not serious(n = 7)	4	3	1.19 (0.22–6.26)	1
Knowledge of MRSA (n = 152)		112	40		
	Yes (150)	110	40	0.36 (0.04–2.67)	0.575
	No (n = 4)	2	2		
Have ever managed MRSA(n = 152)		76	75		
	Yes (*n* = 32)	17	32	2.67 (1.32–5.41)	0.005
	No (*n* = 104)	61	43		
Extent of MRSA problem in hospital (n = 85)		64	21		
	Very serious (n = 25)	20	5	0.68 (0.22–2.13)	0.517
	Serious (*n* = 53)	41	12	0.75 (0.27–2.041)	0.571
	Not serious(n = 7)	3	4	4.78 (0.97–23.47)	0.061
Correct order of resistance in hospital (n = 156)		114	42		
	EMVC (*n* = 17)^a^	8	9	3.61 (1.39–10.11)	0.018
	Others (*n* = 137)	104	33		

*VRE* vancomycin resistant enterococci, *CRE* carbapenem resistant enterococci, *ESBL* extended-spectrum beta-lactamases, *MRSA* methicillin resistant *Staphylococcus aureus*, *KBTH* Korle-Bu Teaching Hospital

^a^EMCV, ESBL-producers>MRSA>CRE > VRE

**Table 4:** Multivariate analysis of factors associated with senior physicians compared to junior physicians

Age	Adjusted OR	*P*-value
Knowledge of CRE	2.35 (1.18–4.93)	0.012
Patients at the hospital are at risk of VRE	1.71 (0.73–3.42)	0.027
Knowledge of VRE	4.63 (1.51–8.22)	0.001
Patients at the hospital are at risk of VRE	3.11 (1.42–5.81)	0.071
Patients at the hospital are at risk of ESBLs	2.33 (1.24–6.11)	0.093
ESBL-producers>MRSA>CRE > VRE	2.06 (1.46–9.81)	0.213
Knowledge of ESBLs	2.61 (1.92–9.81)	0.059
Have previously managed MRSA infections	1.83 (1.01–3.91)	0.067

Adjusted for significant terms (P < 0.05) in bivariable comparisons

*OR* odds ratio, *MRSA* methicillin resistant *Staphylococcus aureus*, *ESBLs* extended-spectrum beta-lactamases, *CRE* carbapenem resistant enterobacteria, *VRE* vancomycin resistant *enterococcus* species

### Associated effects of age on knowledge of CRE and VRE among senior and junior physicians

**Fig. 3:**
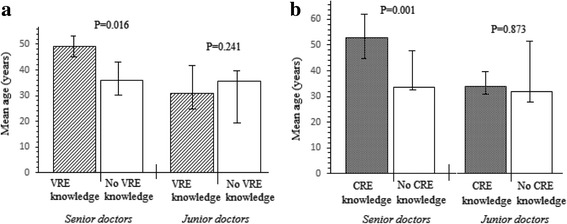
Comparisons of senior and junior physicians regarding the associated effect of age on (**a**) knowledge of VRE. The mean increase in age associated with VRE knowledge was significant among senior physicians; (**b**) knowledge of CRE. The mean increase in age associated with CRE knowledge was significant among senior physicians. Error bars represent 95% confidence intervals of means. Comparisons performed with multiple linear regression using effect size determination — with possible interaction of the variables computed by analysis of covariance (ANCOVA). CRE, carbapenem resistant enterobacteria; VRE, vancomycin resistant *enterococcus* species

The associations between significant terms in the multivariate regression analysis for senior versus junior physicians were further assessed to directly test the significance of age on physicians’ knowledge of VRE and CRE. Figure 3 shows that senior physicians who knew about CRE were significantly younger than those who knew about VRE. The associated increase in mean age observed among senior physicians due to their knowledge of CRE was 10.5 years (95% CI, 8.03–16.73; *P* = 0.016). Similarly, the knowledge of VRE-associated increase in mean age of senior physicians was 16.1 years (95% CI, 13.21–19.22; P = 0.001). Senior physicians with knowledge of VRE were also more likely to know about CRE (OR, 1.21, 95% CI, 2.11–5.93; *P* = 0.010). Among junior physicians, age had no associated effect on their knowledge of CRE or VRE.

## Discussion

It has been suggested that a better understanding of what physicians know about and believe regarding issues of antimicrobial use and resistance can enhance the effectiveness of interventions targeted at improving in-hospital antimicrobial use and control of antibiotic resistance [12]. In this study, conducted in a large Teaching Hospital from a resource limited setting, there was variable knowledge of antibiotic resistance among physicians concerning the causes and scale of antibiotic resistance. The extent of antibiotic resistance in the respondents’ department was likely to have been underestimated. Senior physicians were more likely to have better knowledge of antibiotic resistant bacteria of public health importance compared to junior physicians.

The majority of physicians in this study perceived antibiotic resistance as a global problem while a minority regarded it a problem in their own departments. These findings are similar to studies from other settings where respondents agreed that antibiotic resistance was an important problem but less in their own practice compared to national and the rest of the world [4, 14, 20, 32]. These findings however differ from that in a Brazilian study where most respondents considered antibiotic resistance a problem across all the three levels [16]. It has been suggested that such findings may indicate that respondents see the risks of antibiotic resistance as more theoretical than concrete, and this may weaken efforts at behavior change [32]. These findings can have a potential impact on patient care and infection control activities; as physicians are less likely to pay attention to the antibiotic resistance in selecting antibiotics if they consider it less of a problem in their practice. When asked about the causes of antibiotic resistance, physicians in our study rated overuse of antibiotics in the population and hospital as most important causes of antibiotic resistance and over use in animals as the least important cause. In a similar survey in the Democratic Republic of Congo, respondents rated self-medication and non-completion of antibiotic treatment as the most important factors [20]. In-hospital transmission was rated as the least important cause of antibiotic resistance [20]. In our study, the majority of respondents answered that antibiotics were used appropriately in their units or departments. This finding if true, augurs well for attempts at controlling antibiotic resistance in the hospital. However, it may also reflect a socially desirable answer and will require more rigorous methods to adequately explain. Although a recent study in the hospital has shown relatively low prevalence levels of MRSA [19], nearly all respondents reported knowledge of MRSA. This high percentage of awareness could be due to past outbreaks of MRSA in the hospital (data not published). Also MRSA is cited frequently in literature when antibiotic resistance is discussed making it an easily recognizable mechanism of resistance. The moderate levels of physicians who knew about ESBL is worrying since approximately 50% of all *Escherichia coli* and *Klebsiella* species isolated in the hospital are ESBL producers [24]. This situation may have serious implications for the management of Gram negative infections as well as infection control activities in the hospital.

Knowledge of VRE, MRSA, ESBL, and CRE and their correct order of prevalence in the hospital were found to be associated with being a senior doctor. This finding is at variance with another survey of physicians in which no association was found between level of training and knowledge of antibiotic resistance [14]. Our findings may rather reflect previous experience with antibiotic resistance, a phenomenon which can lead to better recognition of antibiotic resistance as a problem [32]. This may be exemplified by the finding that 52% of senior physicians who expressed knowledge about ESBL also reported having managed an ESBL-related infection versus 21% of junior physicians with knowledge about ESBL and who had managed ESBL-related infections.

Findings from this study suggests the need to increase activities aimed at improving knowledge on antibiotic resistance in the hospital. We suggest that these activities should focus more on junior physicians since they showed inadequate knowledge on antibiotic resistance and are more likely to be prescribers of antibiotics [14]. Hospital educational programmes should include concepts of antibiotic resistance development, multidrug resistant organisms and modalities for preventing the development and spread of antibiotic resistance. Also antibiotic use and resistance concepts should be incorporated into the clinical education programs of medical schools.

This study has some potential limitations. As with most self-administered questionnaires not completed under supervision, we cannot exclude the possibility that respondents verified their answers with other personnel. To minimize this bias, we insisted on independent effort from all study participants. There is the chance that respondents may have given socially desirable answers. It is also possible that the multiple choice format adopted in the questionnaire may have urged respondents’ memory leading to desirable answers. To reduce the chances of receiving social acceptable answers from respondents, no identifiers were attached to the questionnaires to ensure confidentiality and respondents were allowed to answer the questions within their own time and privacy. The survey was a single site study and it may not be possible to extend findings to other healthcare settings. In Ghana, where a majority of antibiotics are prescribed by general practitioners in primary care [33] it will be ideal to conduct further studies among this group of physicians with regards to their knowledge, attitudes and practices concerning antibiotic resistance. This would complement evidence provided in this study to influence and guide future interventions.

## Conclusion

In summary, respondents from this survey showed variable knowledge on the extent and causes of antibiotic resistance; with a high likelihood for underestimating the problem in their own departments or units. To improve antibiotic use and control of antibiotic resistance in the hospital, there is the need to increase education on antibiotic resistance among physicians of this hospital with emphasis on junior physicians.

## Additional file

Additional file 1 Survey instrument. This comprised a 40-item questionnaire for assessing the knowledge, attitudes and perceptions on antibiotic resistance among physicians in Korle-Bu Teaching Hospital. (PDF 1638 kb)

## Abbreviations

AEY: Alfred Edwin Yawson
AKL: Appiah-Korang Labi
ANCOVA: Analysis of covariance
ANOVA: Analysis of variance
AOR: Adjusted Odds ration
CI: Confidence Intervals
CRE: Carbapenem resistant enterobacteria
EMCV: ESBL, MRSA, CRE.VRE
MJN: Mercy Jemima Newman
MRSA: Methicillin resistant *Staphylococcus* species
NAA: Nii Armah Adu-Aryee
NJ: New Jersey
NON: Noah Obeng-Nkrumah
OR: Odds ratio
r: Pearson coefficient
SB: Stephanie Bjerrum
SPSS: Statistical Package for Social Sciences
USA: United States of America
VRE: Vancomycin resistant *Enterococcus* species
*X*^*2*^: Chi-square
YOA: Yaw Ofori-Adjei
