# Supplementary material for: Physicians’ knowledge, attitudes, and perceptions concerning antibiotic resistance: a survey in a Ghanaian tertiary care hospital
Source: BMC Health Serv Res. 2018 Feb 20;18:126. doi: 10.1186/s12913-018-2899-y (PMC5819203; doi:10.1186/s12913-018-2899-y)
Supplement: Supplementary file 1 — Survey instrument. This comprised a 40-item questionnaire for assessing the knowledge, attitudes and perceptions on antibiotic resistance among physicians in Korle-Bu Teaching Hospital. (PDF 1638 kb) [file 12913_2018_2899_MOESM1_ESM.pdf]

Please ensure you have signed the consent form before proceeding to fill the questionnaire

The questionnaire must be filled independently without consultations with anybody

KNOWLEDGE, ATTITUDES AND PERCEPTIONS ON ANTIBIOTIC RESISTANCE AMONG PHYSICIANS IN KORLE-BU TEACHING HOSPITAL

This questionnaire is part of a study to assessing antimicrobial use in the Korle-Bu Teaching Hospital

Kindly complete this questionnaire by clicking the appropriate boxes

Please work independently without consultations or verification

THANK YOU for choosing the fill the questionnaire independently without any help

August 2015

A. Demographics and General Information

1) Age ☐ 2) Sex: Male ☐ Female ☐

3) Department Click or tap here to enter text.

4) Level of training:
a. House officer ☐ b. Medical Officer/Resident ☐ c. Senior resident/Specialist
d. Senior Specialist/Consultant ☐

5) Duration of practice since completion of medical School: Click or tap here to enter text.

B. Perceptions on Antibiotic resistance

6) How will you grade the level of antibiotic resistance worldwide
a. Very important ☐ b. Important c. Not important d. I don't know

7) How will you grade the level of antibiotic resistance in the country
a. Very important ☐ b. Important c. Not important d. I don't know

8) How will you grade the level of antibiotic resistance in the hospital
a. Very important ☐ b. Important c. Not important d. I don't know

9) How will you grade the level of antibiotic resistance in your department
a. Very important ☐ b. Important c. Not important d. I don't know

10) How will you rate the impact of antibiotic resistance on patient safety in your department?
a. Very important ☐ b. Important c. Not important d. I don't know

11) Do you think antibiotics are used appropriately in your department?
a. Yes ☐ b. No ☐

Continue on page 2

You may call or text any of the following numbers for enquiries: 0206300700 0244863056
You may also send an email to guylabi@gmail.com

Please ensure you have signed the consent form before proceeding to fill the questionnaire

The questionnaire must be filled independently without consultations with anybody

KNOWLEDGE, ATTITUDES AND PERCEPTIONS ON ANTIBIOTIC RESISTANCE AMONG PHYSICIANS IN KORLE-BU TEACHING HOSPITAL

This questionnaire is part of a study to assessing antimicrobial use in the Korle-Bu Teaching Hospital

Kindly complete this questionnaire by clicking the appropriate boxes

Please work independently without consultations or verification

THANK YOU for choosing the fill the questionnaire independently without any help

C. Perceptions on causes of antibiotic resistance

To what extent do you think the factors below contribute to the development of antibiotic resistance? Rank them on the scale of 1-3 (where 1 is very important; 2 is moderately important; and 3 is minimally important)

12) Overuse of antibiotics in the hospital: 1. ☐ 2. ☐ 3. ☐

13) Overuse of antibiotics in the population: 1. ☐ 2. ☐ 3. ☐

14) Ineffective antibiotic control in the hospital: 1. ☐ 2. ☐ 3. ☐

15) Use of antibiotics in animals: 1. ☐ 2. ☐ 3. ☐

16) Poor quality antibiotics: 1. ☐ 2. ☐ 3. ☐

17) Too low antibiotic dosages: 1. ☐ 2. ☐ 3. ☐

18) Self-medication: 1. ☐ 2. ☐ 3. ☐

19) Antibiotic treatment not completed: 1. ☐ 2. ☐ 3. ☐

D. Knowledge and attitude on antibiotic resistance of public health importance

20) Do you know about Vancomycin Resistant Enterococcus (VRE)? Yes ☐ No ☐

21) Have you ever managed a patient with VRE? Yes ☐ No ☐

22) Do you think patients in this hospital are risk of VRE? Yes ☐ No ☐

23) Do you think this hospital has a problem with VRE? Yes ☐ No ☐

24) If yes to 23. to what extent? a. Very serious ☐ b. Serious ☐ c. Not serious

Continue on Page 3

You may call or text any of the following numbers for enquiries: 0206300700 0244863056
You may also send an email to guylabi@gmail.com

Please ensure you have signed the consent form before proceeding to fill the questionnaire

The questionnaire must be filled independently without consultations with anybody

KNOWLEDGE, ATTITUDES AND PERCEPTIONS ON ANTIBIOTIC RESISTANCE AMONG PHYSICIANS IN KORLE-BU TEACHING HOSPITAL

This questionnaire is part of a study to assessing antimicrobial use in the Korle-Bu Teaching Hospital

Kindly complete this questionnaire by clicking the appropriate boxes

Please work independently without consultations or verification

THANK YOU for choosing the fill the questionnaire independently without any help

---

25) Do you know about carbapenem resistant enterobacteria (CRE)? Yes ☐ No ☐

26) Have you ever managed a patient with (CRE)? Yes ☐ No ☐

27) Are patients of this hospital at an increased risk of CRE infections? Yes ☐ No ☐

28) Do you think this hospital has a problem with (CRE)? Yes ☐ No ☐

29) If yes to 21. to what extent? a. Very serious ☐ b. Serious ☐ c. Not serious

30) Do you know about extended-spectrum beta-lactamase producing enterobacteria (ESBL)?
Yes ☐ No ☐

31) Have you ever managed an infection caused by an ESBL? Yes ☐ No ☐

32) Do you think patients of this hospital are at risk of infections caused by ESBL? Yes ☐ No ☐

33) Do you think this hospital has a problem with ESBL? Yes ☐ No ☐

34) If yes to 33. to what extent? a. Very serious ☐ b. Serious ☐ c. Not serious

35) Do you know of methicillin resistant Staphylococcus aureus(MRSA)? Yes ☐ No ☐

36) Have you ever managed an infection caused by an MRSA? Yes ☐ No ☐

37) Do you think patients of this hospital are at risk of developing MRSA infections? Yes ☐ No ☐

Continue on page 4

You may call or text any of the following numbers for enquiries: 0206300700 0244863056
You may also send an email to guylabi@gmail.com

Please ensure you have signed the consent form before proceeding to fill the questionnaire

The questionnaire must be filled independently without consultations with anybody

KNOWLEDGE, ATTITUDES AND PERCEPTIONS ON ANTIBIOTIC RESISTANCE AMONG PHYSICIANS IN KORLE-BU TEACHING HOSPITAL

This questionnaire is part of a study to assessing antimicrobial use in the Korle-Bu Teaching Hospital

Kindly complete this questionnaire by clicking the appropriate boxes

Please work independently without consultations or verification

THANK YOU for choosing the fill the questionnaire independently without any help

38) Do you think this hospital has a problem with MRSA? Yes ☐ No ☐

39) If yes to 38. to what extent? a. Very serious ☐ b. Serious ☐ c. Not serious

40) Which do you think is the correct order of prevalence in Ghana:

1. ESBLs > MRSA > VRE > CRE 2. MRSA > ESBLs > CRE > VRE 3. ESBLs > MRSA > CRE < VRE

a. 1 ☐ b. 2 ☐ c. 3 ☐ d. Don't know ☐

You have successfully completed the questionnaire on knowledge, attitudes and perception of physcians on antibiotic resistance in Korle-Bu Teaching Hospital

Safely keep the completed questionnaire. You will be contacted for collection.

THANK YOU FOR PARTICIPATING IN THIS STUDY
